# Prevalence and correlates of disordered eating at a large state university before and after the onset of the COVID-19 pandemic

**DOI:** 10.1186/s40337-024-01056-2

**Published:** 2024-10-01

**Authors:** Carly R Pacanowski, Christine Skubisz, David Borton, Rachel Ryding

**Affiliations:** 1 Department of Health Behavior and Nutrition Sciences, University of Delaware, 26 N College Avenue, Newark, DE 19712 USA; 2 Department of Natural and Applied Sciences, Bentley University, Waltham, MA USA; 3 Center for Drug and Health Studies, University of Delaware, Newark, DE USA; 4 Wake Forest University School of Medicine, Winston-Salem, NC USA

**Keywords:** COVID-19, Disordered eating, Eating disorder, College, Survey, Undergraduate, Screener, Emerging adulthood, Surveillance, Risk factor

## Abstract

**Background:**

Emerging adulthood is a transitory period in which disordered eating (DE) manifests; collecting data on the prevalence of DE among this population as well as demographic and behavioral correlates are important public health goals.

**Methods:**

Data from an annual survey of undergraduate students at a large state university from 2019 to 2022 were analyzed, allowing researchers to compare prevalence and correlates before and after the onset of the COVID-19 pandemic using two brief screeners: the SCOFF and Eating Disorder Screener for Primary Care (ESP). We hypothesized that rates of DE would be greater after the onset of COVID-19 as compared to before. We also hypothesized that those identifying as women, reporting higher alcohol or drug use, and contemplating suicide would have greater odds of reporting symptoms consistent with DE.

**Results:**

DE was significantly lower in pre-pandemic years compared to pandemic years: ESP pre = 38.01%(*n* = 704), pandemic = 48.79%(*n* = 645), *p* < 0.001; SCOFF pre = 22.82%(*n* = 422), pandemic = 31.46%(*n* = 414), *p* < 0.001. Logistic regressions showed women and students who contemplated suicide reported significantly greater DE, regardless of screener or time period. Inconsistent relationships were found between DE and current substance use.

**Conclusion:**

These findings may inform targeted interventions for those most vulnerable to disordered eating.

## Abstract (plain language summary)

It is important to track the rates of disordered eating (DE) in vulnerable populations like emerging adults (those who are between the ages of 18 and 25). Many emerging adults attend college, and during this time DE may occur. The COVID-19 pandemic has led to situations, like being isolated from friends and family, that we assume could increase DE. To present data on the rates of DE before and after the start of COVID-19, researchers collected data on whether college students engaged in DE two years before the start of COVID-19 (2019, 2020) and two after COVID-19 (2021, 2022). They considered whether characteristics of the student, like whether they were a man or woman or their race, were related to DE. DE increased significantly after COVID-19. Using one questionnaire, DE was about 38% before COVID-19 and increased to nearly 50% after COVID-19. Across all four years, women and students who thought about suicide were more likely to report DE. It is important to direct resources to students who are experiencing DE – which we now know could be as many as 50%. DE contributes to health problems and can worsen over time, leading to a life-threatening eating disorder.

## Introduction

The COVID-19 pandemic has negatively affected those with eating disorders (EDs) [1]. Epidemiological surveillance is important to examine changes in both ED and disordered eating (DE) [2] before and after the onset of the pandemic. EDs and DE are correlated with negative health and well-being outcomes including chronic physical health conditions, mental illness, reduced quality of life, and reduced functionality at work or school [3]. EDs are clinically diagnosable mental health conditions [4] with serious consequences, such as impaired ability to perform work, school, and life responsibilities, lessened quality of life, hospitalizations, and suicide attempts [5–7]. While EDs can be severe and result in serious consequences, the prevalence of EDs is relatively low. The most common ED, Binge Eating Disorder, has a reported prevalence of 3.5% in women [8]. DE, which includes cognitions and behaviors occurring in EDs such as being overly concerned with one’s shape and weight, dieting, restriction, and binge eating is common, especially during vulnerable periods of life like emerging adulthood [9]. Emerging adulthood is a distinct developmental stage from adolescence and adulthood and occurs when individuals are between 18 and 25 years old [10]. During emerging adulthood many individuals attend college and live and work within a university campus community. College is a transitory period in which DE patterns manifest; the age at which individuals develop an ED is between the ages of 18 and 21 [8]. Stress related to the transition to college, which could result from new roles and responsibilities related to food procurement, storage, and preparation, living away from the family of origin for the first time, navigating life with roommates, forming romantic partnerships, academic and financial responsibilities, etc., may increase DE. Further exacerbating the situation, the COVID-19 pandemic created situations that increase DE risk including isolation, removal of pre-existing social support systems, increased interpersonal conflict among housemates during social distancing, weight changes, increased use of video communication software that shows self-view, and increased stress, anxiety and depression [11]. Thus, the combination of transitioning to college and the pandemic creates a fertile ground for DE among emerging adults.

Epidemiological surveillance is important for tracking prevalence rates over time and for understanding which subgroups have higher rates of illness [12]. While numerous studies have assessed EDs and DE on college campuses, most use lengthy questionnaires. Disseminators of large survey mechanisms are often wary of adding additional assessment instruments due to increasing participant burden. Brief screening instruments lower participant burden by being short in length and not cognitively demanding to complete when assessing EDs and DE in non-clinical samples. Subgroups can then be the focus of targeted interventions to reduce disease burden for both individuals and society. In the field, two brief and low-burden screeners, the SCOFF [13] and the Eating Disorder Screener for Primary Care (ESP) [14] are available for assessing the prevalence of DE in non-clinical samples.

It is important to note that neither the SCOFF nor the ESP are diagnostic instruments. They are screening tools used to assess prevalence and while the original publications refer to thresholds for ruling in or out EDs or “probable ED”, EDs are diagnosed using other methods, for example, the Eating Disorder Examination Interview [15]. Because the SCOFF and the ESP were developed prior to the DSM-5, the instruments may not detect ED diagnoses other than those for which sensitivity and specificity and associated indices were measured – anorexia nervosa and bulimia nervosa - meaning that prevalence estimates may be for DE and not probable clinical EDs. For this reason, we use the terminology “disordered eating” throughout this manuscript.

Several published studies have used either the SCOFF and/or ESP in community samples of or including emerging adults and suggest that women may have a greater risk for DE. Select studies are included in Table 1.

**Table 1:** Results from select studies that using the SCOFF and/or include emerging women/female adult and participants

Studies using the SCOFF ^a^					
Citation	***n***	**Sample Characteristics**	**Age (years)**	**% Women** **or Female Participants**	**ED Prevalence**
[16]	296	Graduate students, United States (US)	77% of sample *≤* 26 (range = 20–51)	72.4% (*n* = 215)	16.2% (*n* = 48) women more likely to be at risk for ED (*p* < 0.01)
[17]	326	Undergraduate students, US	20.3 ± 1.2 (range = 18–25)	100%	33.6%
[18]	198	University students and community members, Australia	24.8 ± 4.1 (range = 20–35)	71.7% (*n* = 142)	Overall = 12.9% Females = 32.5%
[19] ^b^	77,193	Undergraduate and graduate students, US and Canada	23.1 ± 6.3	65.9%	Overall = 20.3% Females = 24.7%
[20]	2,822	Undergraduate and graduate students, US	98.5% undergraduates 18–25 78.8% graduates 18–30	52.8% (*n* = 1,489)	Higher threshold ^c^ Undergraduate students = 9.4% Female undergraduate students = 13.5% Graduate students = 5.8% Female graduate students = 9.3%
[21]	1,092	Saudi Arabia	23.0 *±* 3.5 (range = 18–30)	100%	41.3%
Studies using the ESP ^d^					
Citation	***n***	**Characteristics**	**Age (years)**	**% Women** **or Female**	**Prevalence**
[22]	150	Participants with Type 1 Diabetes Mellitus	11–25	49%	20.7%
[23]	574	The Netherlands	22.2 *±* 3.1 (range = 18–35)	68.5%	Higher threshold ^e^ 11.7%
[21]	1,092	Saudi Arabia	23.0 *±* 3.5 (range = 18–30)	100%	44.3%

^a^ SCOFF threshold of *≥* 2 abnormal responses based on the original publication [13]

^b^ Several other studies reported prevalence according to the SCOFF, but were also part of the Healthy Minds Study and are not included in this table as the same participants may have been included

^c^ SCOFF threshold of *≥* 3 abnormal responses

^d^ ESP threshold of *≥* 2 abnormal responses based on the original publication [14]

^e^ ESP threshold of *≥* 3 abnormal responses

In these studies, the reported prevalence of disordered eating ranges from less than 10% to almost 45% depending on the sample. Prevalence estimates may vary, in part due to samples being mixed genders, and combined age or developmental groups. It is necessary to understand the prevalence of DE to monitor trends of illness and the impact of interventions. Further, given the effects of the COVID-19 pandemic, it is important to understand how rates of DE may have changed. The primary objective of the present study was to compare the prevalence of DE before and after the onset of the COVID-19 pandemic in a sample of undergraduate students. We hypothesized that the prevalence of DE would be higher after the onset of the pandemic compared to before. The secondary objective of this study was to identify correlates of DE among our sample of students both before and after the onset of the pandemic. We hypothesized that those identifying as women, reporting higher alcohol or drug use, and contemplating suicide would have greater odds of reporting DE. In addition to addressing these hypotheses, we conducted exploratory analyses using demographic, social, and behavioral characteristics to explore whether consistent themes emerged with regard to factors placing individuals at increased odds for DE.

## Materials and methods

The College Risk Behavior Survey (CRBS) is an annual survey of undergraduate students at a large public university in the mid-Atlantic region of the United States that has been conducted since the mid-1990s. Each spring semester, researchers send an email invitation to participate in the survey to a sample of 3,000 undergraduate students currently enrolled on the main campus. A new sample is generated each year. The survey is administered via Qualtrics, takes about 15 min for students to complete, and students are offered a $5 gift card as an incentive for participating [24]. The CRBS measures demographics, substance use, mental health, as well as other health knowledge, health risk behaviors, and social practices of students.

In this study, we examined DE measures over four years of survey data, from 2019 to 2022. In 2019, 1,138 students completed the survey (38% response rate); in 2020, 764 students completed the survey (25% response rate). In 2021, 765 students completed the survey (25% response rate); in 2022, 640 students completed the survey (21% response rate). For this study, data from years 2019 and 2020 are combined and classified as “pre-pandemic”, while data from years 2021 and 2022 were combined and classified as “after the onset of the pandemic”. The CRBS survey is administered each year during the spring semester, typically running from early to middle March to the end of April. The 2020 survey administration was a unique cycle; while the survey was launched in March, that same week the campus enacted its initial shutdown due to the COVID-19 pandemic, at which point students vacated the residence halls and the majority of classes shifted to a virtual format. While the 2020 survey technically remained open to students after the campus shutdown, student engagement with the survey virtually halted. The only survey responses from 2020 that were analyzed for this paper were collected prior to the campus shutdown when student life was still relatively normal. Lower survey response rates in 2020, 2021, and 2022 compared to 2019 are likely attributable to pandemic-related disruptions in that influenced student engagement.

### Demographics

The study included the following key demographic variables: gender^1^ (*woman, man*); race/ethnicity (*Non-Hispanic Black, Hispanic*, *Non-Hispanic Asian, Non-Hispanic Another Race/Multiple Races (this includes Middle Eastern, Native Hawaiian or Pacific Islander, Alaska Native or American Indian, or other or biracial/multiracial, Non-Hispanic White)*, year in college *(first year, second year, third year, fourth year and beyond);* and place of residence *(home with family, off campus, on campus)*.

### Social characteristics and behavioral health

The CRBS survey measured binary variables related to student life and behavioral health. These variables included sorority or fraternity membership (*yes/no*); participation in intercollegiate or intramural athletic teams (*yes/no*); past month marijuana use *(yes/no);* past month alcohol use *(yes/no)*; and whether students seriously considered suicide in the past year *(yes/no).*

Since 2019, the CRBS has also included two brief screeners for EDs, described below. We selected these two screeners after consulting the Academy for Eating Disorders’ Epidemiology and Public Health Practice Special Interest Group.

### SCOFF

The SCOFF was designed to be a brief screening tool with a simplistic scoring algorithm [13]. The SCOFF asks the respondent to answer yes/no to five items, each associated with a letter in the acronym SCOFF (**S**ick, **C**ontrol, **O**ne, **F**at, **F**ood).

1. Do you make yourself **S**ick because you feel uncomfortably full?
2. Do you worry that you have lost **C**ontrol over how much you eat?
3. Have you recently lost more than **O**ne stone (14 lb.) in a three-month period?
4. Do you believe yourself to be **F**at when others say you are thin?
5. Would you say that **F**ood dominates your life?

Per the original publication, answering “yes” to two or more items provided 100% sensitivity and 87.5% specificity for anorexia nervosa and bulimia nervosa. A 2020 systematic review and meta-analysis of 25 studies reported a pooled sensitivity of 86% and specificity of 83% [25].

### ESP

Another brief screener, the Eating Disorder Screener for Primary Care [14], was created for both use in primary care and college samples. It contains four questions:

1. Are you satisfied with your eating patterns?
2. Do you ever eat in secret?
3. Does your weight affect the way you feel about yourself?
4. Do you currently suffer with or have you ever suffered in the past with an eating disorder?

The original publication reported 100% sensitivity and 71% specificity for two or more abnormal responses (an abnormal response is considered a “no” to question 1, “yes” to questions 2–4) as a threshold for having an ED.

### Analysis

Chi-squared analyses were used to compare the prevalence of DE before versus after the onset of the pandemic. Two logistic regression models were used to test our hypotheses regarding correlates of DE, one regression model using the SCOFF score to define the dependent variable and one regression model using the ESP score. The dependent variable was DE, defined by the original publications of the SCOFF and the ESP as *≥* 2 abnormal responses. An abnormal response is considered “yes” to any item on either questionnaire, except for item 1 for the ESP, in which case “no” is an abnormal response. The number of abnormal responses per individual was tallied separately for each questionnaire, and the proportion of individuals with at least two abnormal responses was calculated.

Substance use, biological sex, athlete, and fraternity/sorority membership were coded using dummy variable binary responses; whereas, race, living situation, and class year were categorical variables. Analyses were conducted using Stata v14. The logistic command in Stata was used, which produces maximum-likelihood dichotomous logistic models. Alpha was set as *p* *≤* 0.05. For each respective model (SCOFF and ESP), all independent variables were entered at the same step.

## Results

### Overall demographics

Over four years (2019, 2020, 2021, and 2022), a total of 3,308 students were surveyed. Survey data from 2019 to 2020, before the onset of the pandemic, constituted 57% of the total data. Table 2 displays the core demographics of survey respondents by survey year. While most demographic variables were consistent across study years, in 2021 there were fewer first year students and students living on campus. This decline is likely attributable to overall trends in fewer students taking classes in person and living on campus during this pandemic year.

**Table 2:** Key demographic variables by year

Demographic Variable	2019 (*n* = 1,138)	2020 (*n* = 764)	2021 (*n* = 765)	2022 (*n* = 641)	Total (*n* = 3,308)
Woman	70.35%	68.94%	71.24%	66.98%	69.58%
Man	29.65%	31.06%	28.76%	33.02%	30.42%
Non-Hispanic White	74.95%	76.51%	75.82%	67.14%	73.82%
Non-Hispanic Black	6.10%	4.86%	3.27%	7.36%	5.40%
Hispanic	7.60%	7.61%	7.06%	8.29%	7.61%
Non-Hispanic Asian	8.84%	8.01%	9.54%	10.80%	9.19%
Other/Mixed Race	3.01%	3.02%	4.31%	6.42%	3.97%
First Year	30.84%	28.57%	13.86%	24.80%	25.21%
Second Year	25.81%	25.03%	26.14%	22.15%	25.00%
Third Year	21.85%	27.79%	33.46%	28.24%	27.15%
Fourth Year or older	21.50%	18.61%	26.54%	24.80%	22.64%
Living on campus	56.06%	51.05%	26.67%	43.68%	45.71%
Living at home with family	6.68%	8.38%	15.82%	12.17%	10.25%
Living off campus	37.26%	40.58%	57.52%	44.15%	44.04%

Additionally, over a quarter (*n* = 945, 28.7%) of respondents were members of fraternities or sororities and about a quarter (*n* = 805, 24.4%) participated in athletics. About two-thirds of respondents consumed alcohol at least monthly (*n* = 2,226, 67.6%) and almost one-quarter reported using marijuana at least monthly (*n* = 763, 23.2%). Finally, 311 respondents (9.5%) reported contemplating suicide in the past year. The remaining respondents did not report contemplating suicide within the past year or did not respond to this question (*n* = 3,139, 90.5%).

### Prevalence of DE before and after the onset of the COVID-19 pandemic

**Fig. 1:**
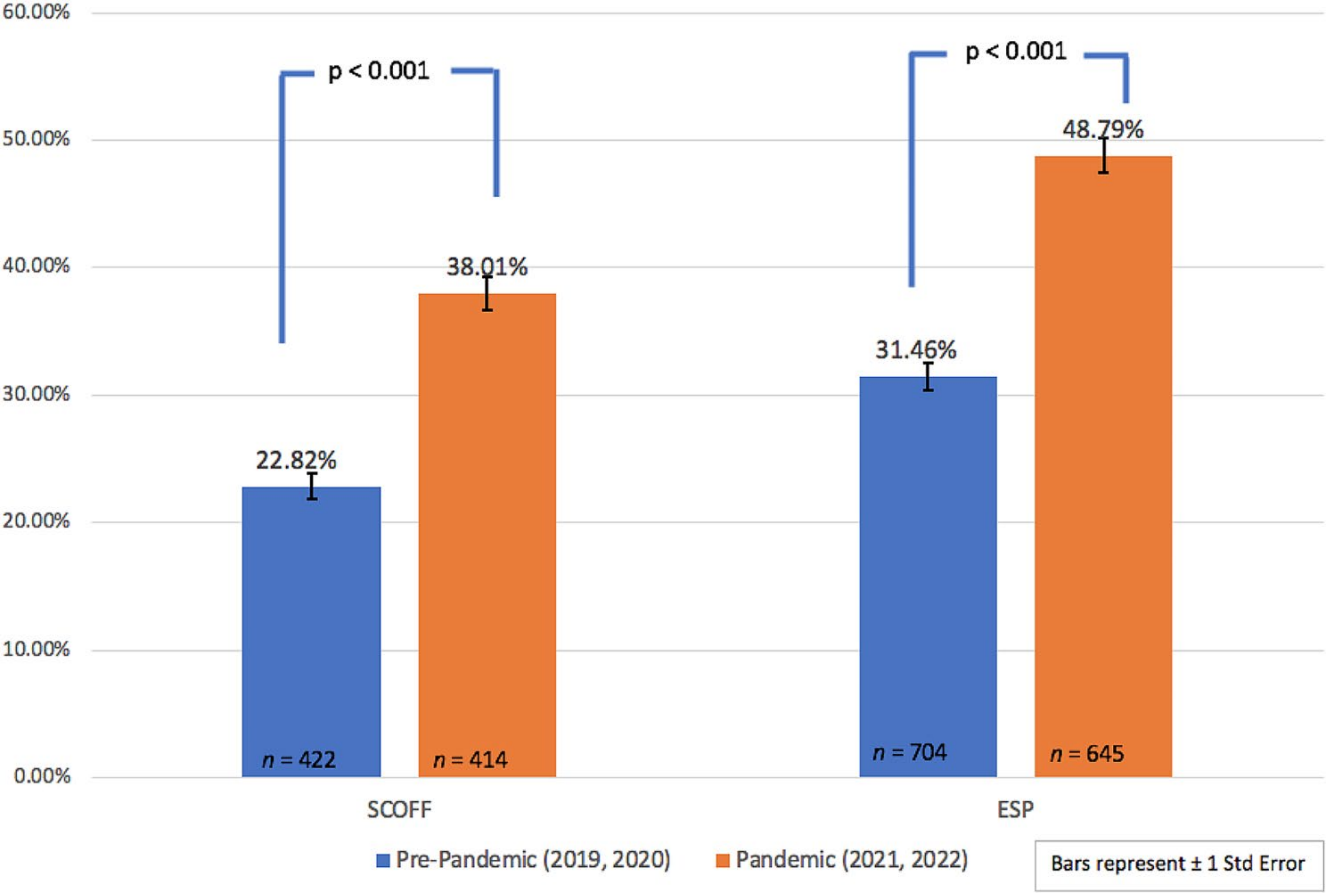
Percent of students reporting DE before and after the onset of COVID-19 by screener

Figure 1 displays the percentage of students reporting DE before the onset of the pandemic versus after the onset of the pandemic. Before the onset of the pandemic, 22.82% (*n* = 422) of respondents met the threshold for DE according to the SCOFF questionnaire, and after the onset of the pandemic, 31.46% (*n* = 414) of respondents met the threshold for DE (*X*^2^ = 29.50, *p* < 0.001). Using the ESP, before the onset of the pandemic, 38.01% (*n* = 704) of students reported DE, and after the onset of the pandemic, 48.79% (*n* = 645) of students reported DE (*X*^2^ = 36.66, *p* < 0.001).

Table 3 provides the percentage of respondents providing an abnormal response to individual items in each screener. The prevalence of abnormal responses is provided by gender and for the total sample. For most of the items, a pattern is evident. Women report an abnormal response at a greater prevalence than men, and the prevalence of the total sample indicating an abnormal response increases with time.

**Table 3:** Percentage and number of individuals reporting abnormal^1^ SCOFF and ESP responses, individual indicators, and aggregate scores, by gender (%, n), 2019–2022

	2019			2020^2^			2021			2022		
	Men *n* = 333	Women *n* = 790	Total *n* = 1,123	Men *n* = 223	Women *n* = 526	Total *n* = 763	Men *n* = 202	Women *n* = 545	Total *n* = 765	Men *n* = 163	Women *n* = 426	Total *n* = 636
SCOFF												
1. Do you make yourself sick because you feel uncomfortably full?	7.60%, 25	9.94%, 78	9.25%, 103	6.25%, 13	13.02%, 66	11.13%, 81	9.19%, 17	16.35%, 85	14.42%, 104	12.34%, 19	19.66%, 81	17.46%, 106
2. Do you worry you have lost control over how much you eat?	11.52%, 38	27.39%, 215	22.69%, 253	6.67%, 14	24.12%, 123	19.35%, 142	14.52%, 27	32.63%, 170	28.35%, 205	16.23%, 25	31.80%, 131	28.22%, 171
3. Have you recently lost more than one stone (14 lb.) in a three month period?	10.61%, 35	7.26%, 57	8.25%, 92	5.77%, 12	8.43%, 43	7.79%, 57	8.74%, 16	10.19%, 53	9.87%, 71	11.76%, 18	8.74%, 36	9.57%, 58
4. Do you believe yourself to be fat when others say you are thin?	18.79%, 62	41.69%, 326	34.89%, 388	11.96%, 25	40.67%, 207	32.38%, 237	25.27%, 47	47.69%, 248	41.55%, 300	23.38%, 36	48.05%, 197	41.16%, 249
5. Would you say that food dominates your life?	6.38%, 21	14.30%, 112	11.96%, 133	5.24%, 11	12.94%, 66	10.76%, 79	6.45%, 12	20.42%, 106	17.20%, 124	7.84%, 12	20.63%, 85	17.13%, 104
Total with *≥* 2 abnormal responses	13.11%, 43	28.55%, 223	23.99%, 266	7.32%, 15	26.48%, 134	21.13%, 153	15.85%, 29	37.23%, 191	31.88%, 227	17.76%, 27	36.10%, 148	31.11%, 187
ESP												
1. Are you satisfied with your eating patterns?	25.76%, 85	42.29%, 332	37.40%, 417	28.57%, 60	47.27%, 242	41.85%, 308	34.78%, 64	46.45%, 242	43.83%, 316	28.76%, 44	46.00%, 190	42.43%, 258
2. Do you ever eat in secret?	13.03%, 43	21.58%, 169	19.05%, 212	6.25%, 13	16.47%, 84	13.52%, 99	16.76%, 31	25.72%, 134	23.82%, 172	14.94%, 23	26.65%, 109	24.5%, 148
3. Does your weight affect the way you feel about yourself?	47.58%, 157	70.11%, 549	63.43%, 706	40.0%, 84	63.46%, 323	56.62%, 415	57.30%, 106	74.90%, 391	70.12%, 507	55.19%, 85	69.27%, 284	66.01%, 400
4. Do you currently suffer with or have you ever suffered in the past with an eating disorder?	6.69%, 22	16.71%, 131	13.75%, 153	5.71%, 12	14.31%, 73	11.99%, 88	10.81%, 20	26.30, 137	22.85%, 165	9.15%, 14	32.36%, 133	26.73%, 162
Total with *≥* 2 abnormal responses	23.71%, 78	44.62%, 348	38.41%, 426	21.15%, 44	43.37%, 219	37.14%, 270	36.41%, 67	53.56%, 278	49.37%, 355	29.61%, 45	53.56%, 218	48.17%, 289

^1^ An abnormal response is considered “yes” to any item except for item 1 for the ESP, in which case “no” is an abnormal response

^2^ Starting in 2020, the number of men and women does not add up to equal the total *n* because additional response options (i.e., nonbinary, self-description) were included. Students selecting responses other than man or woman are factored into the total student population but are too small as an analytical category to include without compromising anonymity

### Logistic regression results

Tables 4 and 5 display results from logistic regression models using DE according to the SCOFF and ESP, respectively, as the dependent variable. Independent variables were gender, race, sorority/fraternity membership, athletic participation, class year, place of residence, past month alcohol use, past month marijuana use, and suicide consideration in the past year. For logistic regression models, all mean Variance Inflation Factors were less than 2.

**Table 4:** Logistic regression model with prevalence of DE as the Dependent Variable (2 or more abnormal responses on the SCOFF) pre and Post COVID-19 pandemic

Variable (comparator)	Pre-pandemic (2019, 2020)				Pandemic (2021, 2022)			
	Odds ratio	SE	CI (95%)	*p*-value	Odds ratio	SE	CI (95%)	*p*-value
**Gender**								
Women (men)	3.03**	0.484	2.22–4.15	0.000	2.63**	0.412	1.93–3.58	0.000
**Race/ethnicity**								
N-H Black (N-H white)	0.53*	0.167	0.29–0.98	0.044	0.71	0.222	0.39–1.31	0.279
Hispanic (N-H white)	0.87	0.202	0.55–1.37	0.555	1.12	0.266	0.71–1.78	0.627
N-H Asian (N-H white)	0.86	0.203	0.55–1.37	0.535	0.81	0.187	0.51–1.27	0.357
N-H Another Race/Multiple Races (N-H white)	1.01	0.359	0.50–2.03	0.972	0.64	0.199	0.35–1.18	0.155
**Year in college**								
Second year (first year)	0.96	0.166	0.68–1.35	0.806	1.15	0.241	0.77–1.74	0.492
Third year (first year)	0.90	0.183	0.61–1.34	0.616	1.14	0.248	0.74–1.74	0.557
Fourth year (first year)	0.99	0.211	0.66–1.52	0.985	1.20	0.273	0.77–1.88	0.414
**Residence**								
Home with family (on campus)	0.74	0.202	0.42–1.26	0.264	0.70	0.155	0.45–1.08	0.107
Off campus (on campus)	1.34	0.224	0.97–1.86	0.076	0.68*	0.117	0.49–0.95	0.025
**Greek life (sorority/fraternity)**								
Yes (no)	0.89	0.120	0.67–1.16	0.403	0.96	0.139	0.72–1.27	0.758
**Athlete (intercollegiate, club, intermural sports)**								
Yes (no)	0.87	0.124	0.65–1.15	0.316	1.05	0.167	0.78–1.44	0.730
**Alcohol (past month)**								
Yes (no)	1.19	0.183	0.88–1.61	0.252	1.46*	0.221	1.08–1.96	0.013
**Marijuana (past month)**								
Yes (no)	1.66**	0.229	1.26–2.17	0.000	1.02	0.168	0.74–1.41	0.888
**Contemplated suicide (past year)**								
Yes (no)	4.38**	0.822	3.03–6.32	0.000	3.47**	0.672	2.37–5.07	0.000

*Note* SE = Standard Error; N-H = Non-Hispanic; * *p* < 0.05; ** *p* < 0.01

**Table 5:** Logistic regression model with prevalence of DE as the Dependent Variable (more than 2 abnormal responses on the ESP) pre and Post COVID-19 pandemic

Variable (comparator)	Pre-pandemic (2019, 2020)				Pandemic (2021, 2022)			
	Odds ratio	SE	CI (95%)	*P*-value	Odds ratio	SE	CI (95%)	*P*-value
**Gender**								
Women (men)	2.53**	0.318	1.99–3.26	0.000	2.15**	0.293	1.65–2.81	0.000
**Race/ethnicity**								
N-H Black (N-H white)	0.76	0.179	0.47–1.19	0.228	0.55*	0.159	0.31–0.97	0.040
Hispanic (N-H white)	0.82	0.161	0.55–1.19	0.288	1.26	0.289	0.81–1.98	0.306
N-H Asian (N-H white)	0.99	0.188	0.67–1.43	0.482	0.59*	0.124	0.39–0.89	0.012
N-H Another Race/Multiple Races (N-H white)	0.82	0.251	0.44–1.48	0.482	0.76	0.204	0.45–1.28	0.298
**Year in college**								
Second year (first year)	1.25	0.183	0.94–1.67	0.131	1.27	0.249	0.86–1.87	0.227
Third year (first year)	1.36	0.228	0.96–1.87	0.088	1.59*	0.325	1.07–2.38	0.023
Fourth year (first year)	1.38	0.246	0.96–1.95	0.083	1.51	0.322	0.99–2.29	0.051
**Residence**								
Home with family (on campus)	0.70	0.157	0.46–1.09	0.116	0.74	0.148	0.49–1.09	0.126
Off campus (on campus)	0.96	0.136	0.73–1.27	0.772	0.61**	0.099	0.44–0.84	0.002
**Greek life (sorority/fraternity)**								
Yes (no)	0.74*	0.088	0.59–0.94	0.012	0.99	0.137	0.76–1.31	0.763
**Athlete (intercollegiate, club, intermural sports)**								
Yes (no)	0.71**	0.087	0.56–0.90	0.005	1.05	0.155	0.79–1.41	0.723
**Alcohol (past month)**								
Yes (no)	1.27	0.164	0.99–1.64	0.057	1.38*	0.192	1.05–1.81	0.021
**Marijuana (past month)**								
Yes (no)	1.24	0.155	0.97–1.59	0.081	1.58 **	0.248	1.16–2.14	0.004
**Contemplated suicide (past year)**								
Yes (no)	5.11**	1.031	3.43–7.58	0.000	4.42**	0.987	2.85–6.85	0.000

*Note* N-H = Non-Hispanic; * *p* < 0.05; ** *p* < 0.01

Consistent with our hypotheses, women had significantly higher odds of DE (ranging from 2.15 to 3.03) than men regardless of questionnaire or time period (all *p* < 0.01). Those who contemplated suicide within the past year had the highest odds of DE of any variable measured in this study. Odds ranged from 3.47 to 5.12 depending on the questionnaire and time period (all *p* < 0.01).

Regarding substance use, with the SCOFF as the dependent variable, those who consumed alcohol at least monthly had 1.46 higher odds of DE compared to those who consumed alcohol less than monthly (*p* < 0.05) after the onset of the pandemic, but the relationship was not significant before the onset of the pandemic. Conversely, those who consumed marijuana at least monthly had 1.66 higher odds of DE compared to those who consumed marijuana less than monthly (*p* < 0.01) before the onset of the pandemic, but this relationship was not significant after the onset of the pandemic. Using the ESP as the dependent variable, there were significantly higher odds of DE for those consuming alcohol at least monthly (1.38, *p* < 0.05) and marijuana at least monthly (1.58, *p* < 0.01) compared to those consuming the substance less frequently after the onset of the pandemic but not before.

While there are some significant relationships between demographic and social/behavioral characteristics and DE, a pattern in which both screeners show the same association, during the same time period, is not evident.

## Discussion

This study adds to the growing body of literature on the health effects of the COVID-19 pandemic by analyzing data collected before and after its onset. Statistically significant differences were found in the prevalence of DE before versus after the onset of the COVID-19 pandemic according to two well-known brief screening instruments, the SCOFF and the ESP, in a sample of emerging adult college students. While the prevalence of DE was concerning before the onset of the pandemic, with 22.82% and 31.46% of students scoring as experiencing DE using the SCOFF and ESP, respectively, these prevalence rates climbed to 38.01% and 48.79% in the pandemic years. Pre-pandemic rates were in line with those found in prior studies of this population (with some studies showing rates between 16.2% and 33.6% [16–19], but the rates found post-pandemic were greater than those found in previous studies.

Subgroups consistently at greater risk for DE included women and those contemplating suicide regardless of screening questionnaire or measurement time period. Other research using the SCOFF has found more women score as having DE both before and after the onset of the pandemic [3, 18, 26]. A large body of literature links higher rates of suicide and thoughts about suicide to EDs [27].

Alcohol and marijuana use had inconsistent relationships with DE, but when significant, greater use was associated with higher odds of DE. While the present study did not evaluate clinical substance use disorders, there is also a vast literature linking substance use to EDs: a recent metanalysis showed that 21.9% of individuals with an ED also had a substance use disorder in their lifetime, with alcohol and marijuana as the third and fifth most commonly used substances, respectively [28]. Thus, many of the findings from the present study are in line with the extant literature.

Limitations of the present study must be addressed. The SCOFF and ESP are screening tools for predicting DE risk and do not provide prevalence rates for EDs within the surveyed population. Additionally, the screening tools used have not been validated in racial or ethnic minority groups. Out of 25 studies included in a meta-analysis and systematic review of the SCOFF [25], only four reported race and ethnicity. Thus, the present study adds data on DE risk and race and ethnicity to the extant literature. Confounding variables may have made the sample in 2021 and 2022 different from the sample in 2019 and 2020 in ways that were not measured. COVID-19 led to changes in the way people live: people were less likely to leave their homes and interact with others in person, and more likely to communicate virtually. It is possible that loneliness, social isolation, and grief impacted the rates of DE in 2021 and 2022. Finally, while the CRBS survey was sent to a sample of 3,000 undergraduates each year, in 2019–2022, the response rate ranged from 21 to 38%. Thus, results presented in this manuscript may be affected by non-response bias if those who opted not to complete the survey were systematically different than those who completed the survey.

DE prevalence rates were higher using the ESP compared to the SCOFF. This may indicate differences in sensitivity between the two tools. A new tool was proposed in a recent study [29] for detecting EDs in primary care and combines items from the SCOFF and other well-known ED questionnaires and may be of use for future research. In addition, surveillance mechanisms such as the CRBS should be instated or continue to assess prevalence of ED/DE and change over time in responses to implemented interventions or societal shifts.

## Conclusion

This study analyzed data from an annual survey of undergraduate students at a large state university from 2019 to 2022 to compare the prevalence of and correlates of DE before and after the onset of the COVID-19 pandemic. DE was found to be significantly lower in pre-pandemic years compared to pandemic years. Particularly interesting was the finding that women and students who contemplated suicide had significantly greater ED risk, regardless of screener or time period. Inconsistent relationships were found between residence, current substance use, and risk of disordered eating. These findings may inform targeted interventions for those most vulnerable to DE, especially in potential future situations that resemble the COVID-19 pandemic.

## Data Availability

The data are not publicly available due to student privacy concerns within the institution supporting the research.

## Footnotes

1 **Note.** More response categories were offered for gender (*Transgender Woman or Feminine, Transgender Man or Masculine, and Gender non-binary / Gender non-conforming, as well as options to self-describe or not answer*). However, for these groups, sample sizes were too small to maintain anonymity. Thus, results from categories with sufficient sample sizes are presented.

## Abbreviations

ED: Eating Disorder
DE: Disordered Eating
ESP: Eating Disorder Screen for Primary Care
